# Relaxin‐2 Ameliorates Spinal Cord Injury by Inhibiting Microglia Activation

**DOI:** 10.1002/kjm2.70041

**Published:** 2025-05-20

**Authors:** Ji‐Huan Wang, Hong‐Biao Sheng, Jun‐Kun Li

**Affiliations:** ^1^ Department of Orthopedics Fengcheng Hospital of Fengxian District Shanghai People's Republic of China

**Keywords:** AQP4, BBB, M2 microglia activation, Relaxin‐2, spinal cord injury (SCI)

## Abstract

This study aims to assess the therapeutic effectiveness of Relaxin‐2 (RLN‐2) in promoting functional recovery and neuroprotection following spinal cord injury (SCI) in mice. Furthermore, continuous subcutaneous infusion of Serelaxin (0.5 mg/kg/day; human recombinant relaxin‐2) improved neurological recovery, as evidenced by higher Basso‐Beattie‐Bresnahan (BBB) scores and reduced foot‐stepping angles compared to the SCI group. Additionally, RLN‐2 effectively reduced edema in the injured spinal cord, as shown by decreased water content and downregulated AQP4 expression at mRNA and protein levels. RLN‐2 reduced oxidative stress markers such as malondialdehyde (MDA) and reactive oxygen species (ROS) and increased the activity of catalase (CAT). Further, RLN‐2 mitigated neuroinflammation by reducing the levels of pro‐inflammatory cytokines (TNF‐α and IL‐6) and by inhibiting the activation of M1 microglia while promoting the polarization of M2 microglia. It also inhibited the activation of the NF‐κB signaling and strengthened the activation of the STAT6 signaling in the spinal cord of SCI mice. These findings suggest that RLN‐2 may be a promising therapeutic agent for the treatment of spinal cord injury.

## 1 Introduction

Spinal cord injury (SCI) arises due to trauma to the spinal cord from multiple causes, causing motor and sensory impairments (encompassing both deep and cutaneous sensation) alongside autonomic nervous system dysfunction distal to the injury site [1]. Current epidemiological studies report that global annual SCI incidence exceeds 500,000 new cases, with cumulatively affected populations surpassing 15 million worldwide [2]. This condition induces profound neural tissue damage, leading to different levels of motor and sensory deficits, including complete loss of physical function in severe cases [3]. Therapeutic interventions for SCI remain problematic; while stem cell‐based approaches demonstrate potential in preclinical models, their clinical translatability requires additional verification regarding safety and effectiveness [4].

The pathophysiology of SCI is complex, involving both primary and secondary injuries. Primary injury occurs due to direct external force on the spinal cord, while secondary injury includes inflammatory responses, oxidative stress (OS), and cell apoptosis [5]. After SCI, OS increases reactive oxygen species (ROS), damaging cell membranes and exacerbating injury through lipid peroxidation [6]. Simultaneously, microglia, lymphocytes, and neutrophils mediate an inflammatory response, releasing pro‐inflammatory cytokines that worsen tissue damage and disrupt the blood‐spinal cord barrier [7]. OS and inflammation synergistically amplify nerve damage, while modulating these responses can mitigate secondary injury and enhance recovery [8].

Traumatic damage, inflammation, infections, and other pathologic states cause rapid activation of microglia, the principal immune effector cells found in the middle and peripheral nervous systems, therefore displaying bidirectional functional capabilities [9]. These cell types become different phenotypic states: M1 or M2 categories, which are respectively pro‐inflammatory and anti‐inflammatory. M1‐polarized microglia secrete pro‐inflammatory cytokines such as TNF‐α and IL‐1β, exacerbating neuroinflammation and neuronal damage; M2‐polarized cells produce anti‐inflammatory components that help in tissue remodeling and neuroprotection [10, 11]. Particularly with their activities in controlling inflammatory cascades and oxidative stress responses, which have biphasic polarization patterns, recent studies have pointed to microglial modulation as a new goal for therapeutic development related to SCI. Novel treatment routes for enhancing outcomes of spinal cord injury are influencing the M1/M2 polarization balance of microglia [12, 13].

Relaxin‐2, an insulin‐like peptide hormone chiefly produced by the corpus luteum, was first recognized for its role in pregnancy, specifically in enabling the relaxation of the pubic symphysis and softening the uterine musculature [14]. Recently, Relaxin‐2 has garnered attention for its promising antioxidant and anti‐inflammatory properties [15, 16]. Relaxin‐2 binds RXFP1, reducing vascular inflammation and inflammatory cell infiltration [15], attenuates OS in cardiomyocytes under hypoxia‐reoxygenation by enhancing reduced glutathione utilization [16], and reduces neuronal apoptosis post‐periventricular hemorrhage via the ERK‐nNOS‐NO pathway [17]. This study aims to evaluate the therapeutic potential of Relaxin‐2 in treating spinal cord injury (SCI) by assessing its impact on the M1/M2 microglial polarization profile, thereby suggesting a novel clinical strategy for SCI treatment.

## 2 Materials and Methods

### 2.1 Ethics Statement

This study was approved by the institutional ethics committee (No. 202293588).

### 2.2 Animals

Eight‐ to ten‐week‐old female C57BL/6J mice (18–22 g body weight) were obtained from the Shanghai Laboratory Animal Center (SLAC). All experimental procedures complied with institutional animal care guidelines.

### 2.3 Spinal Cord Injury (SCI) Model

Under aseptic conditions, mice received intraperitoneal anesthesia via 1% sodium pentobarbital injection. A rongeur was used to excise the T9 spinous process, followed by laminectomy to fully expose the spinal cord. Animals were then placed on the IH‐0400 spinal cord impactor, ensuring horizontal alignment of the exposed cord. SCI induction involved applying 50 kdyn to the target site. Successful modeling was validated by observing injury site hemorrhage, hind‐limb spastic contractions, and tail flick reflexes. Sham controls underwent identical laminectomy without spinal cord trauma.

### 2.4 Experimental Groups

Three groups were established: Sham (*N* = 10), SCI (*N* = 10), and SCI + Relaxin‐2 (*N* = 10). The SCI + Relaxin‐2 group received a continuous subcutaneous infusion of Serelaxin (1.7 mg/mL in saline) at a rate to achieve 0.5 mg/kg/day using osmotic mini‐pumps (Alzet Model 2004, CA, USA) for 28 days [18]. The Sham and SCI groups received continuous subcutaneous saline infusion using the same method. All animals in each group were used for all measurements, including lesion area analysis, spinal cord water content measurement, ELISA, RT‐PCR, and western Blot, ensuring consistent and comprehensive data collection.

### 2.5 Basso‐Beattie‐ Bresnahan (BBB) Locomotor Rating Scale

Motor function was assessed immediately post‐injury (day 0), and then weekly on days 7, 14, 21, and 28 using the BBB scoring system and locomotor testing. Assessments were conducted by investigators blinded to the experimental groups. The BBB scoring system [19], which ranges from 0 to 21 points with higher scores indicating better motor function, was used to evaluate motor function recovery.

### 2.6 Locomotor Function Testing

Motor performance was evaluated using a custom‐built murine walking beam (“beam”) [16]. Behavioral assessments included baseline measurements (pre‐training) and post‐injury evaluations on days 7, 14, 21, and 28 post‐SCI. A Panasonic NV‐DS12 camera (25 frames/s) recorded trials for subsequent analysis. Footstep angles (FSA) were measured from selected video frames using Image Tool 2.0 software.

### 2.7 Lesion Area Volume Assessment

On day 28 post‐SCI, mice were euthanized with CO_2_ inhalation and transcardially perfused with PBS followed by 4% formaldehyde. Spinal cord segments from T7 to T9 were isolated, cryoprotected, sectioned at 25 μm using a cryostat, and mounted on SuperFrost Plus slides. Every fifth section was stained with Cresyl violet for Nissl staining. Lesion areas in each section were quantified using Image‐Pro Plus 6.2 software at ×2.5 magnification.

### 2.8 Spinal Cord Water Content Measurement

Mice were euthanized with CO_2_ inhalation, and a 0.5 cm segment of spinal cord tissue centered at T9 was weighed (wet weight). After drying at 70°C for 2–3 days (reduced to 37°C overnight), the dry weight was recorded. Water content was calculated as (wet weight‐dry weight)/wet weight × 100%.

### 2.9 Enzyme‐Linked Immunosorbent Assay (ELISA)

Spinal cord tissues were homogenized and centrifuged to isolate supernatants for ELISA analysis. Levels of catalase (CAT), high mobility group box protein 1 (HMGB‐1), interleukin‐6 (IL‐6), and tumor necrosis factor‐alpha (TNF‐α) were measured using commercial kits (Mlbio, China) following the manufacturer's instructions.

### 2.10 CAT Activity Measurement

First, spinal cord tissues were homogenized in ice‐cold phosphate buffer (50 mM, pH 7.0) and centrifuged at 10,000 × g for 10–15 min at 4°C to collect the supernatant containing catalase. Subsequently, the reaction mixture was prepared by combining 1.9 mL of phosphate buffer, 0.1 mL of sample supernatant, and 1.0 mL of freshly prepared hydrogen peroxide (H_2_O_2_, 30 mM) in a microplate well. The absorbance of the reaction mixture was recorded immediately at 240 nm using a spectrophotometer, with readings taken at 0, 30, 60, and 90 s to monitor the rate of H_2_O_2_ decomposition. A blank containing phosphate buffer and H_2_O_2_ (without sample) was used for calibration.

### 2.11 Malondialdehyde (MDA) Determination

MDA levels in spinal cord tissues were quantified using a spectrophotometric thiobarbituric acid (TBA) assay with commercial kits (Mlbio, China).

### 2.12 Dihydroethidium (DHE) Staining

Spinal cord sections were deparaffinized, incubated with DHE staining solution at 37°C for 30 min, and imaged using a confocal microscope (Nikon, Japan). DHE fluorescence intensity was analyzed using Image‐Pro Plus 6.0 software.

### 2.13 Reverse Transcription Polymerase Chain Reaction (RT‐PCR)

Total RNA was extracted from spinal cord samples using TRIzol reagent, and cDNA synthesis was performed with the PrimeScript kit (Takara, Japan). Quantitative real‐time PCR was carried out using the Hieff qPCR SYBR Green Master Mix (YEASEN, China) to determine target gene expression levels, employing the 2^−ΔΔCT^ method for data analysis.

### 2.14 Western Blot Analysis

Protein extraction from spinal cord tissues was performed using RIPA buffer, and protein concentration was quantified via the BCA method. Fifty micrograms of protein per well were separated by SDS‐PAGE, transferred to PVDF membranes, and incubated with primary antibodies against AQP4, CD11b, CD163, p‐IκBα, IκBα, p‐NF‐κB, and p‐STAT6 p65 (all diluted 1:1000; β‐actin diluted 1:2000; Abcam, USA) overnight at 4°C. Secondary antibodies (1:3000; Abcam, USA) were applied for 2 h. Band intensities were visualized using ECL chemiluminescence and quantified with ImageJ software.

### 2.15 Immunohistochemistry

Spinal cord tissues underwent antigen retrieval in EDTA buffer to expose epitopes. Sections were blocked with 5% normal serum to minimize non‐specific binding and permeabilized using 0.1% Triton X‐100 to enhance antibody penetration. The primary antibody AQP4 (Cell Signaling Technology, USA) was diluted in blocking solution, applied, and incubated overnight at 4°C or for 2 h at room temperature, followed by washing to remove unbound antibodies. HRP‐conjugated secondary antibodies were then applied and incubated for 1 h at RT, followed by additional washing. DAB substrate solution was applied to develop color, and the reaction was stopped with distilled water.

### 2.16 Statistical Analysis

Data were analyzed using SPSS 20.0 software, with results presented as mean ± SD, and compared by One‐Way ANOVA, with *n* = 10 per group for each assay. Differences were considered statistically significant at *p* < 0.05.

## 3 Results

### 3.1 Relaxin‐2 Improved Neurological Recovery in SCI Mice

**FIGURE 1:**
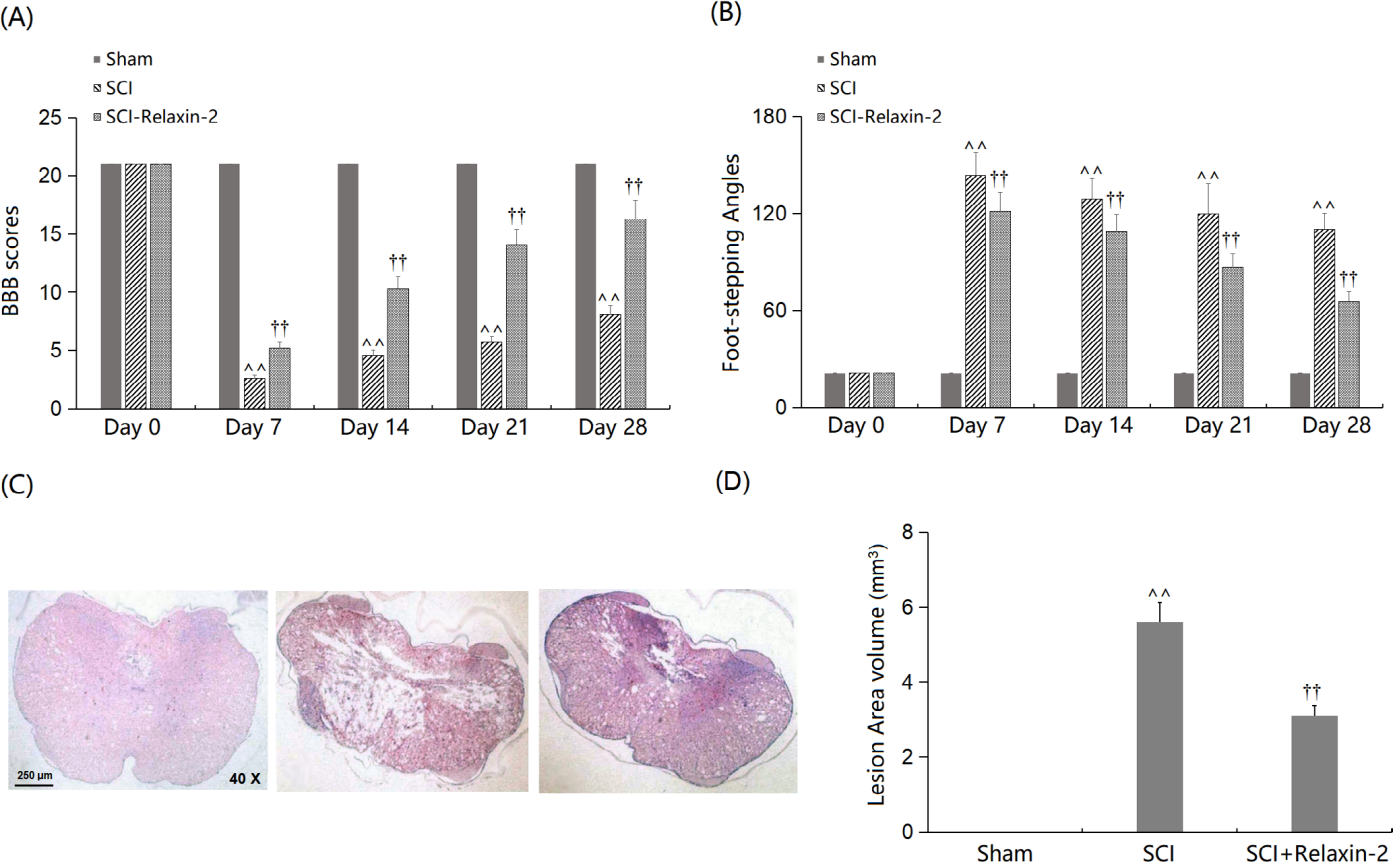
Relaxin‐2 improved neurological recovery in SCI mice. Mice were divided into three groups: Sham, SCI, and SCI + Relaxin‐2. (A) BBB scores on Day 0, Day 7, Day 14, Day 21, and Day 28 were recorded; (B) Foot‐stepping Angles on Day 0, Day 7, Day 14, Day 21, and Day 28 were recorded; (C) Representative Cresyl violet‐stained images of spinal cord lesion areas at 28 days post‐injury; (D) Lesion volume (mm^3^) quantified from spinal cord sections at 28 days post‐injury. (^^, *p* < 0.01 vs. Sham group; ††, *p* < 0.01 vs. SCI group).

Sham, SCI, and SCI + Relaxin‐2 groups were established for animal experiments. On days 7, 14, 21, and 28 post‐modeling, BBB scores were substantially diminished in SCI mice, and this reduction was markedly improved by Relaxin‐2 treatment (Figure 1A). Additionally, the markedly increased FSAs observed in SCI mice on days 7, 14, 21, and 28 post‐modeling were significantly attenuated by Relaxin‐2 (Figure 1B). At 28 days post‐injury, Cresyl violet‐stained spinal cord sections showed larger lesion areas in SCI mice compared to Sham, with reduced lesion areas in Relaxin‐2‐treated mice (Figure 1C). Lesion volume (mm^3^), quantified from these sections, was significantly increased in SCI mice versus Sham (*p* < 0.01) but markedly reduced by Relaxin‐2 treatment (*p* < 0.01 vs. SCI; Figure 1D). Its reduction by Relaxin‐2 reflects attenuated tissue loss, supporting the compound's neuroprotective effects.

### 3.2 Relaxin‐2 Ameliorated Edema in the Spinal Cord of SCI Mice

**FIGURE 2:**
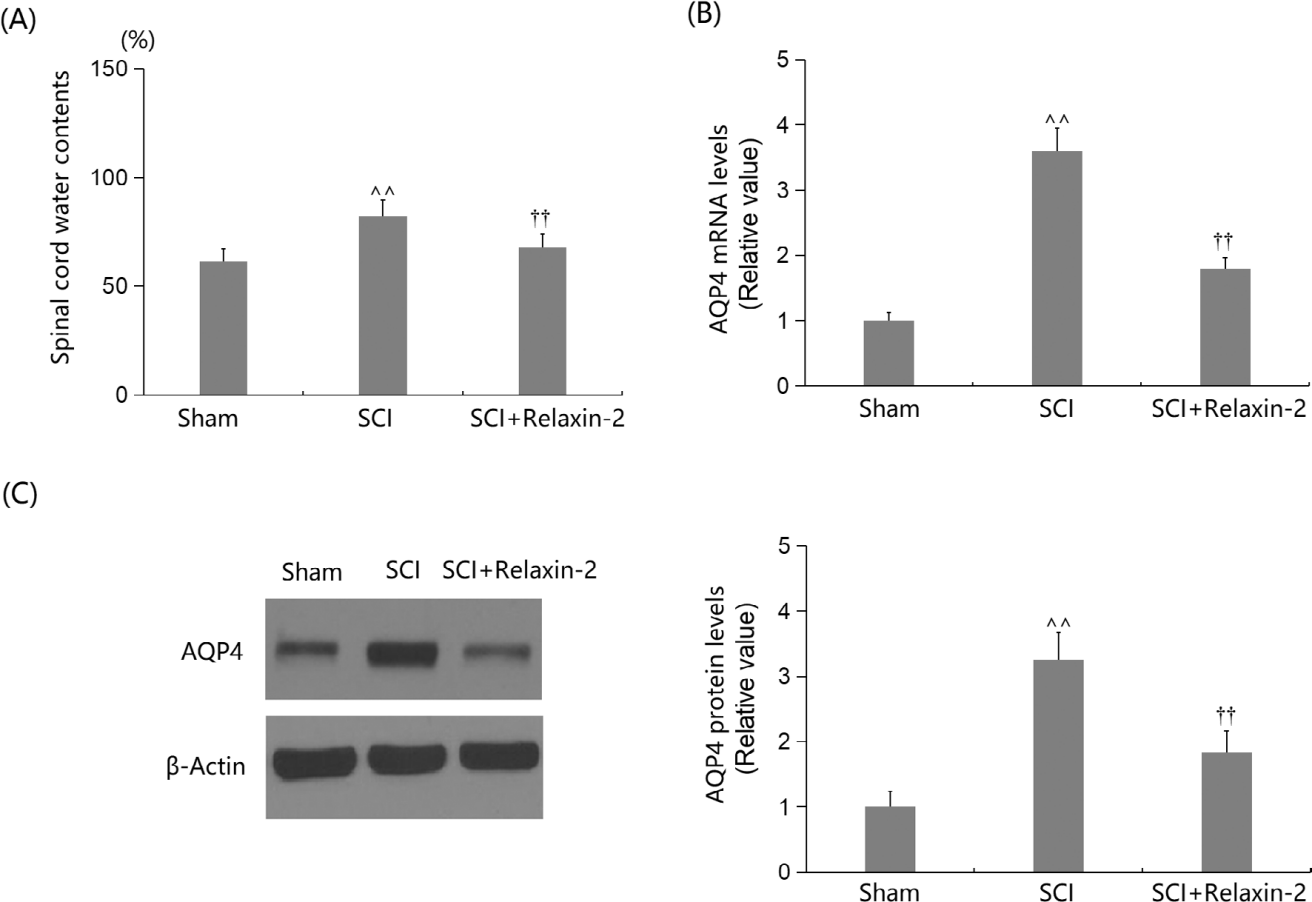
Relaxin‐2 ameliorated edema in the spinal cord of SCI mice. Mice were divided into three groups: Sham, SCI, and SCI + Relaxin‐2. (A) Spinal cord water contents 28 days after SCI; (B) Relative *AQP4* mRNA expression in spinal cord tissues from Sham, SCI, and SCI + Relaxin‐2 treated mice, measured by real‐time PCR; (C) Western blot analysis of AQP4 protein expression in spinal cord tissues from Sham, SCI, and SCI + Relaxin‐2 treated mice. β‐Actin was used as a loading control; (D) Relative protein levels of AQP4 (^^, *p* < 0.01 vs. Sham group; ††, *p* < 0.01 vs. SCI group).

The water content in spinal cord tissues (SCTs) increased substantially from 61.3% to 82.2% in SCI mice, and this increase was markedly reduced to 67.8% by Relaxin‐2 treatment (Figure 2A). Furthermore, the significantly upregulated AQP4 expression in SCTs of SCI mice was remarkably suppressed by Relaxin‐2, as shown by real‐time PCRs and western blotting analysis (Figure 2B,C; Supporting Informations).

### 3.3 Relaxin‐2 Alleviated Oxidative Stress (OS) in SCI Mice

**FIGURE 3:**
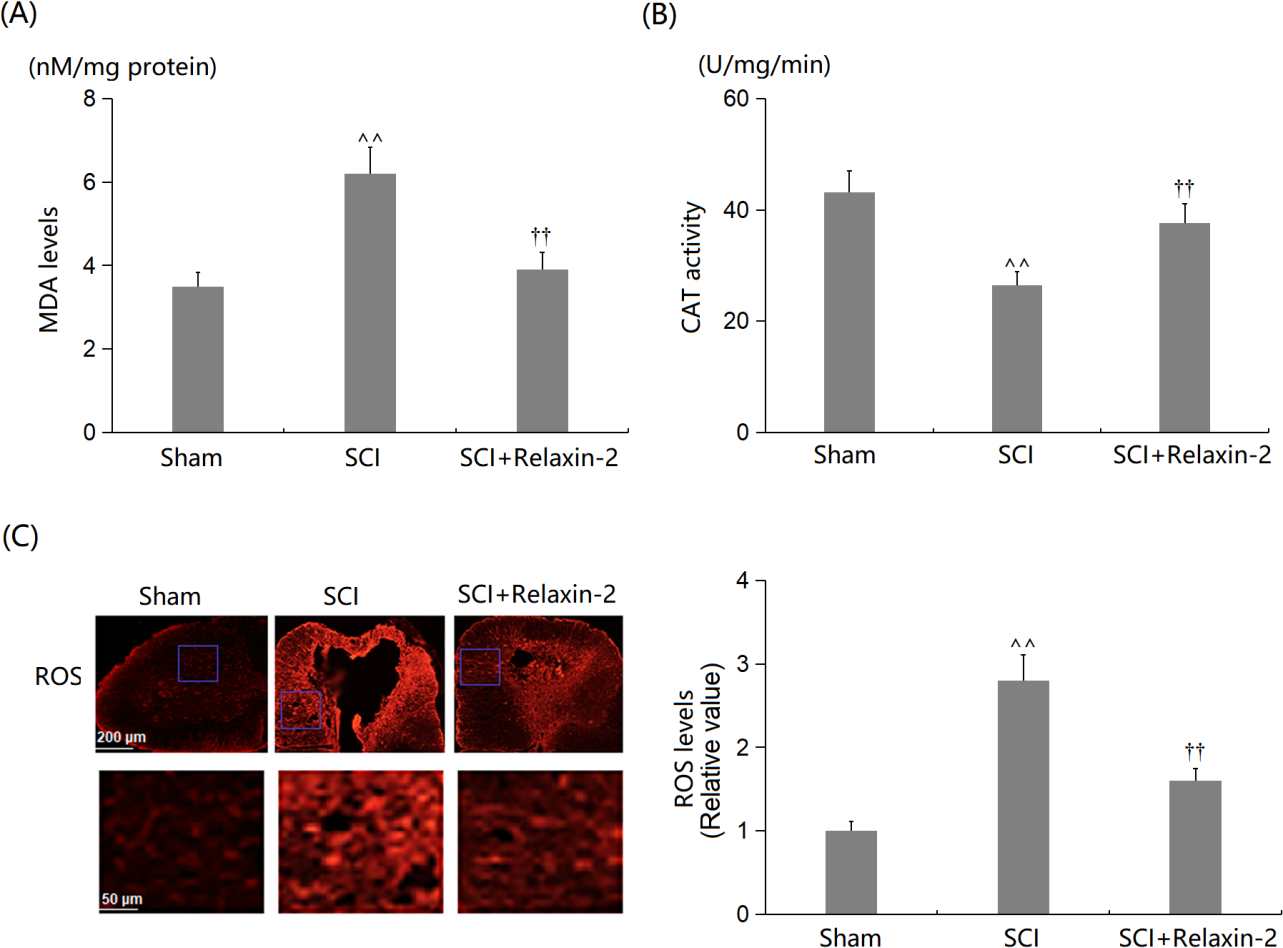
Relaxin‐2 alleviated oxidative stress in SCI mice. Mice were divided into three groups: Sham, SCI, and SCI + Relaxin‐2. (A) MDA levels; (B) CAT activity; (C) ROS levels in gray matter as measured by DHE fluorescence microscopy. Scale bar = 50 μm. The *Y*‐axis represents ROS fluorescence intensity (relative value) (^^, *p* < 0.01 vs. Sham group; ††, *p* < 0.01 vs. SCI group).

MDA levels in SCTs increased from 3.5 to 6.2 nM/mg protein in SCI mice, and this increase was considerably diminished to 3.9 nM/mg protein by Relaxin‐2 (Figure 3A). Additionally, CAT activities in the Sham, SCI, and SCI + Relaxin‐2 groups were 43.2, 26.5, and 37.6 U/mg/min, respectively (Figure 3B). Furthermore, the significantly elevated ROS levels in SCTs of SCI mice were substantially reduced by Relaxin‐2 (Figure 3C).

### 3.4 Relaxin‐2 Suppressed Inflammatory Response in SCTs of SCI Mice

**FIGURE 4:**
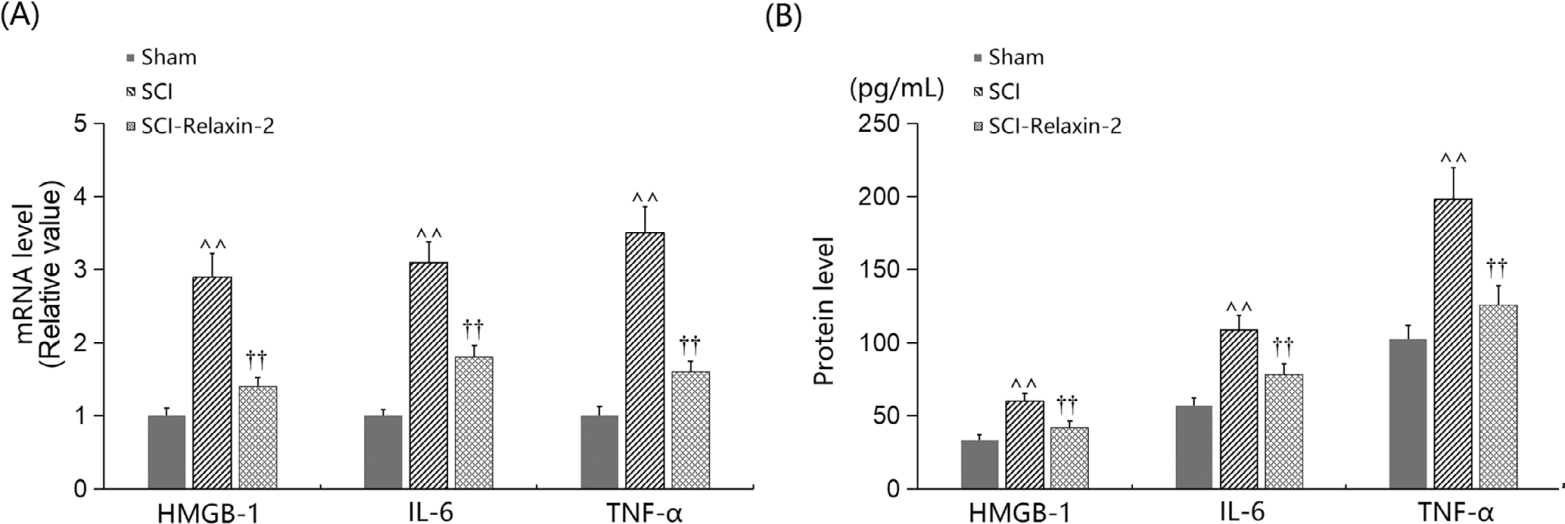
Relaxin‐2 suppressed inflammatory response in the spinal cord of SCI mice. Mice were divided into three groups: Sham, SCI, and SCI + Relaxin‐2. (A) mRNA of HMGB‐1, IL‐6, and TNF‐α as measured by real time PCR; (B) Protein of HMGB‐1, IL‐6, and TNF‐α as measured by ELISA (^^, *p* < 0.01 vs. Sham group; ††, *p* < 0.01 vs. SCI group).

Levels of HMGB‐1, IL‐6, and TNF‐α were significantly upregulated in SCI mice, and these increases were significantly reduced by Relaxin‐2 (Figure 4A). Specifically, HMGB‐1 levels in SCTs increased from 33.5 to 59.8 pg/mL in SCI mice, and this increase was significantly reduced to 42.1 pg/mL by Relaxin‐2. IL‐6 levels in the Sham, SCI, and SCI + Relaxin‐2 groups were 57.1, 108.9, and 78.3 pg/mL, respectively. Similarly, TNF‐α levels in SCTs increased from 102.6 to 198.3 pg/mL in SCI mice, which were significantly reduced to 125.7 pg/mL by Relaxin‐2 (Figure 4B).

### 3.5 Relaxin‐2 Suppressed M1 Microglia Activation While Stimulating M2 Microglia Activation in SCI Mice

**FIGURE 5:**
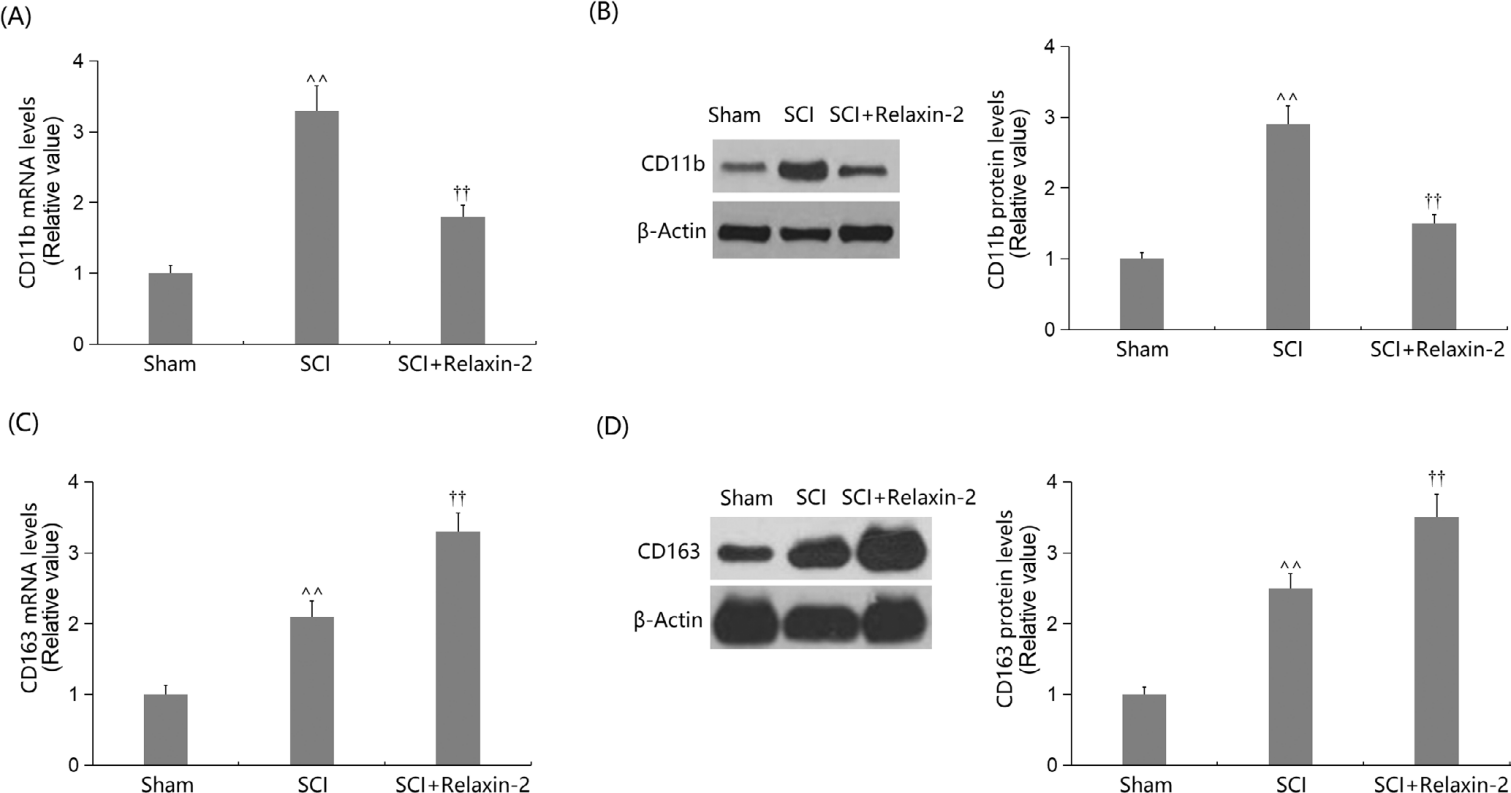
Relaxin‐2 suppressed M1 microglia activation while stimulating M2 microglia activation in SCI mice. (A) mRNA levels of M1 microglia marker CD11b; (B) protein levels of M1 microglia marker CD11b; (C) mRNA levels of M2 microglia marker CD163; (D) Protein levels of M2 microglia marker CD163 (^^, *p* < 0.01 vs. Sham group; ††, *p* < 0.01 vs. SCI group).

CD11b expression, a marker of M1 microglia, was significantly increased in SCI mice, and this elevation was notably repressed by Relaxin‐2 (Figure 5A,B). Conversely, CD163 expression, a marker of M2 microglia, was significantly increased in SCI mice and further dramatically increased by Relaxin‐2 (Figure 5C,D).

### 3.6 Relaxin‐2 Inhibited Activation of the NF‐κB Signaling Pathway in SCTs of SCI Mice

**FIGURE 6:**
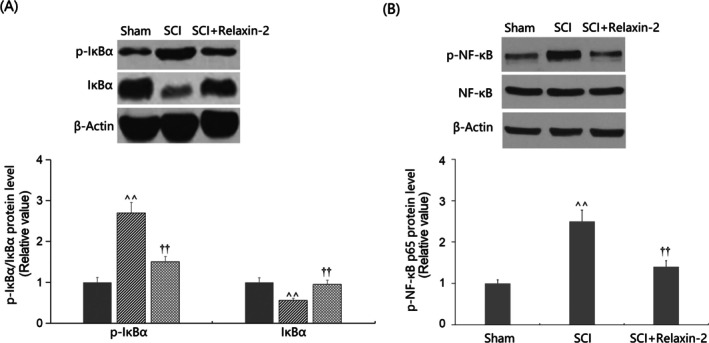
Relaxin‐2 inhibited activation of the NF‐κB signaling in the spinal cord of SCI mice. (A) The levels of p‐IκBα and IκBα; (B) The levels of p‐NF‐κB p65 (^^, *p* < 0.01 vs. Sham group; ††, *p* < 0.01 vs. SCI group).

The NF‐κB pathway, known to drive M1 polarization [20], was activated in SCI mice, as indicated by increased p‐IκBα levels and decreased total IκBα levels; these changes were reversed by Relaxin‐2. These changes were considerably reversed by Relaxin‐2 (Figure 6A). Consistent with this inhibitory effect on NF‐κB signaling, RLN‐2 treatment also significantly reduced p‐NF‐κB p65 expression in SCI mice (Figure 6B; Supporting Informations).

### 3.7 Relaxin‐2 Promoted Activation of the STAT6 Signaling Pathway in SCTs of SCI Mice

**FIGURE 7:**
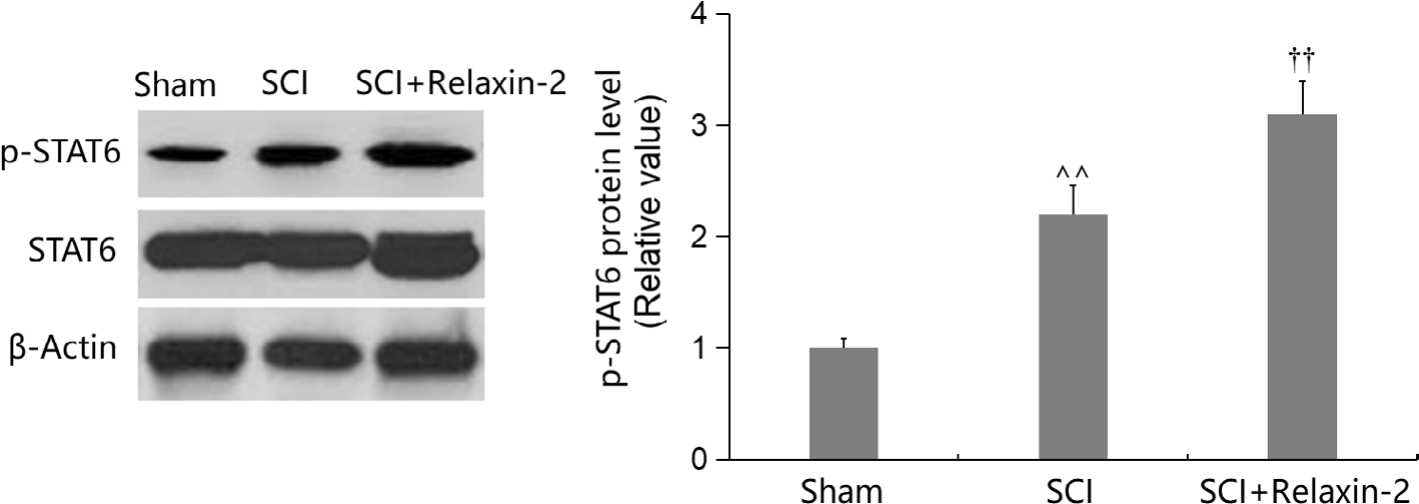
Relaxin‐2 promoted activation of the STAT6 signaling in the spinal cord of SCI mice. The levels of p‐STAT6 were measured by western blot analysis (^^, *p* < 0.01 vs. Sham group; ††, *p* < 0.01 vs. SCI group).

The STAT6 signaling pathway is reported to induce M2 microglial polarization [21]. In this study, p‐STAT6 levels in SCTs were significantly elevated in SCI mice and were further sharply increased by Relaxin‐2 (Figure 7).

## 4 Discussion

Following SCI, motor nerve impairment primarily results from interrupted nerve signaling and disruption of neuromuscular junctions (NMJs) [22]. SCI blocks communication between the spinal cord and peripheral nerves, causing apoptosis of motor nerve terminals, decreased neurotransmitter release, and eventual muscle atrophy [23]. SCI additionally disrupts neuromuscular junction (NMJ) integrity, further worsening motor dysfunction [22]. SCT edema represents another prevalent pathological feature post‐SCI. Edema formation involves two primary mechanisms: vascular and cytotoxic processes. Vascular edema occurs due to blood‐spinal cord barrier breakdown, leading to extracellular fluid accumulation. Conversely, cytotoxic edema arises from intracellular water retention, causing cellular swelling [24]. This edema not only exerts mechanical pressure on spinal cord neural structures but also exacerbates nerve injury [5]. Consistent with Manthou's findings in rats [25], SCI mice exhibited neurological deficits, locomotor impairments, increased lesion volume, and aggravated SCT edema, all of which were markedly reduced by Relaxin‐2 treatment, indicating its protective role in SCI animal models.

SCI induces secondary damage primarily through OS and inflammatory responses [26]. OS triggers excessive ROS and free radical production, impairing cellular membranes and organelle integrity, which ultimately worsens neuronal injury [27]. Inflammatory responses, primarily initiated by microglia activation, trigger the release of multiple pro‐inflammatory factors when these cells become activated, damaging the blood–spinal cord barrier and causing neuronal apoptosis [28]. Liu et al. reported that oxidative stress and inflammatory activation in spinal cord tissues (SCTs) of SCI animals were significantly reduced by Relaxin‐2 treatment, suggesting this compound may mitigate SCI through suppression of OS and inflammation [28].

M1‐polarized microglia typically adopt a pro‐inflammatory phenotype, marked by the secretion of multiple pro‐inflammatory cytokines [29]. These activated cells exacerbate neuroinflammation and contribute to neuronal injury. Under pathological conditions, M1‐polarized microglia express elevated levels of marker proteins like CD11b, a member of the integrin family widely used to identify microglia and macrophages [30]. CD11b expression increases further during microglial activation, correlating with their functional state [31]. Li et al. reported M1 polarization in SCI mice, indicated by increased CD11b expression in SCTs, which was significantly reduced by Relaxin‐2 treatment, suggesting its inhibitory effect on M1 polarization [32].

NF‐κB, a transcription factor, resides in the cytoplasm bound to its inhibitor IκBα [33]. IκBα blocks NF‐κB nuclear translocation by masking its nuclear localization signal. Upon exposure to external stimuli (e.g., inflammatory factors or pathogens), IκBα becomes phosphorylated by IκB kinase (IKK), leading to its ubiquitination and degradation. This releases NF‐κB, allowing nuclear entry and activation of target genes [34]. NF‐κB promotes M1 microglial polarization by regulating inflammation‐related gene expression [35]. In this study, SCI mice exhibited activated NF‐κB signaling in SCTs, which was strongly suppressed by Relaxin‐2, suggesting the compound inhibits M1 polarization via NF‐κB pathway modulation.

M2‐polarized microglia exhibit anti‐inflammatory and tissue‐repair functions, typically mitigating inflammation through secretion of cytokines like IL‐10 and TGF‐β [36]. This phenotype is associated with specific marker proteins, with CD163 serving as a key identifier [37]. CD163, a scavenger receptor involved in hemoglobin/heme clearance, shows marked expression during anti‐inflammatory states [38]. Li et al. reported M2 polarization in SCI mice as part of the injury response, with Relaxin‐2 treatment further augmenting this effect, suggesting its role in promoting M2‐mediated anti‐inflammatory activity [32].

STAT6 signaling is recognized as a critical regulator of M2 microglial polarization. For instance, low‐intensity pulsed ultrasound (LIPUS) promotes M2 polarization via STAT1/STAT6/PPARγ pathway activation, reducing inflammation [39]. Similarly, Loureirin B shifts microglia from M1 to M2 phenotypes through STAT6 signaling, protecting against cerebral ischemia–reperfusion injury [40]. In this study, Relaxin‐2 strongly activated STAT6 signaling in SCI mice, indicating a potential mechanism for its M2‐promoting effects. Future investigations will explore this regulatory pathway using in vitro models to clarify Relaxin‐2's role in microglial polarization.

Future research should assess RLN‐2's translational potential in medical applications and investigate its underlying molecular mechanisms. Given their likely effects on spinal cord injury results, further research should also explore possible interactions of RLN‐2 with estrogen signaling. This would help us to create sex‐specific therapy plans based on a better knowledge of the therapeutic potential of RLN‐2.

## 5 Conclusions

In summary, Relaxin‐2 mitigated spinal cord injury (SCI) in mice by regulating the M1/M2 polarization balance of microglia. Relaxin‐2 suppressed the pro‐inflammatory M1 phenotype, marked by reduced CD11b expression and inhibition of the NF—κB signaling pathway, while promoting the anti‐inflammatory M2 phenotype, indicated by increased CD163 expression and activation of the STAT6 signaling pathway. This bidirectional regulation of microglial polarization not only attenuated neuroinflammation and oxidative stress but also enhanced tissue repair and functional recovery after SCI. These results demonstrate Relaxin‐2's potential as a therapeutic agent targeting microglial polarization to alleviate secondary injury and improve SCI outcomes. Future research should focus on elucidating its detailed molecular mechanisms and evaluating its clinical translatability in preclinical models.

## Conflicts of Interest

The authors declare no conflicts of interest.

## Supporting information


**Data S1.** Supporting Information.

## Data Availability

The data that support the findings of this study are available from the corresponding author upon reasonable request.
