# Supplementary material for: Relaxin‐2 Ameliorates Spinal Cord Injury by Inhibiting Microglia Activation
Source: Kaohsiung J Med Sci. 2025 May 20;41(9):e70041. doi: 10.1002/kjm2.70041 (PMC12412555; doi:10.1002/kjm2.70041)
Supplement: Supplementary file 1 — Data S1. Supporting Information. [file KJM2-41-e70041-s001.pdf]

动物实验伦理审查表 编号（Issue No.）： IACUC No. 202293588
The Table of Animal Experimental Ethical Inspection

一、申请人填写

（一）申请人基本信息

申请人	联系电话	邮箱	部门
王继欢（Jihuan Wang）	+86-021-57522565	happywang625@126.com	骨科 (Department of Orthopedics)
项目负责人	联系电话	邮箱	部门
王继欢(Jihuan Wang)	+86-021-57522565	happywang625@126.com	骨科 (Department of Orthopedics)

（二）项目基本信息

项目名称	项目编号	项目来源
一种新型神经保护剂对脊髓损伤的治疗作用研究	FCH-2022-002	医院基金

（三）供试品信息

供试品名称及编号	是否有毒
新型神经保护剂，编号：NP-2022-08	否

（四）实验人员基本信息

姓名	专业	动物实验工作内容	上岗证号
王继欢 (Jihuan	骨外科	研究新型神经保护剂对脊髓损伤的治疗作用	BMRC-2020-003-ZS

姓名	专业	动物实验工作内容	上岗证号
Wang)			

（五）拟使用动物信息

动物来源	生产许可证编号	品种/品系	等级	数量	雌/雄	体重	周龄
上海实验动物中心	SYXK（沪）2019-0003	C57BL/6J	SPF级	30只	雌	20 - 25 g	8 - 10周

（六）拟实验时间

进驻时间	结束时间
2023 年 1 月	2024 年 1 月

（七）实验动物饲养情况

实验动物饲养设施	使用许可证编号	设施地址	饲养环境	饲养人员
上海实验动物中心	SYXK（沪）2019-0003	上海实验动物中心	屏障环境	实验人员负责

（八）实验内容概述

实验方法、给药途径、观察指标等

实验方法、给药途径、观察指标等	
实验方法	实验需健康雌性 C57BL/6 小鼠 30 只，分为 3 组，每组 10 只：对照组、模型组、药物处理组。采用脊髓损伤模型，通过连续皮下输注新型神经保护剂（0.5 mg/kg/天）来研究其对脊髓损伤的治疗作用。

实验方法、给药途径、观察指标等	
观察指标	BBB 评分、步态角、脊髓组织含水量、AQP4 表达、氧化应激指标（MDA、CAT、ROS）、炎症因子（HMGB-1、IL-6、TNF-α）以及 microglia 的 M1/M2 极化状态等。

是否会造成动物不适或疼痛以及相应处理方法

是否会造成动物不适或疼痛以及相应处理方法	
是	脊髓损伤模型的建立和手术操作可能会给动物带来不适或疼痛。处理方法包括使用适当的麻醉和镇痛药物，确保手术操作熟练，减少动物的应激和痛苦。

若实验含外科程序，请简述手术麻醉方法、剂量、给药方式与术后照顾

项目	
手术名称/方法	脊髓损伤模型建立
麻醉前处理	无
麻醉给药	1% sodium pentobarbital 注射
剂量	0.1 mL/10 g 体重
术后处理	包括适当的镇痛和抗感染措施，确保动物术后恢复良好。

实验结束后动物处理方法描述

实验结束后动物处理方法描述	
实施安乐死	采用 CO_2 吸入法实施安乐死。

实验结束后动物处理方法描述	
(Euthanasia Method)	

（九）声明

声明人	王继欢（签名） 王继欢
日期	2022 年 6 月 1 日

二、伦理委员会填写

（一）审查内容

审查内容	是否符合
是否符合 3Rs 原则	符合
是否符合动物福利“五大自由”原则	符合
实验操作中是否符合实验动物伦理原则	符合
是否充分考虑从业人员的安全要求	符合

（二）审查结果

伦理委员会审批意见	同意
伦理委员会签（章）(Signature/Stamp)：	上海市奉城医院伦理委员会
